# Synthetic five-wave mixing in an integrated microcavity for visible-telecom entanglement generation

**DOI:** 10.1038/s41467-022-33914-5

**Published:** 2022-10-20

**Authors:** Jia-Qi Wang, Yuan-Hao Yang, Ming Li, Haiqi Zhou, Xin-Biao Xu, Ji-Zhe Zhang, Chun-Hua Dong, Guang-Can Guo, C.-L. Zou

**Affiliations:** 1 CAS Key Laboratory of Quantum Information, University of Science and Technology of China, 230026 Hefei, China; 2 CAS Center For Excellence in Quantum Information and Quantum Physics, University of Science and Technology of China, 230026 Hefei, China

**Keywords:** Quantum optics, Nonlinear optics

## Abstract

Nonlinear optics processes lie at the heart of photonics and quantum optics for their indispensable role in light sources and information processing. During the past decades, the three- and four-wave mixing (*χ*^(2)^ and *χ*^(3)^) effects have been extensively studied, especially in the micro-/nano-structures by which the photon-photon interaction strength is greatly enhanced. So far, the high-order nonlinearity beyond the *χ*^(3)^ has rarely been studied in dielectric materials due to their weak intrinsic nonlinear susceptibility, even in high-quality microcavities. Here, an effective five-wave mixing process (*χ*^(4)^) is synthesized by incorporating *χ*^(2)^ and *χ*^(3)^ processes in a single microcavity. The coherence of the synthetic *χ*^(4)^ is verified by generating time-energy entangled visible-telecom photon pairs, which requires only one drive laser at the telecom waveband. The photon-pair generation rate from the synthetic process shows an estimated enhancement factor over 500 times upon intrinsic five-wave mixing. Our work demonstrates a universal approach of nonlinear synthesis via photonic structure engineering at the mesoscopic scale rather than material engineering, and thus opens a new avenue for realizing high-order optical nonlinearities and exploring functional photonic devices.

## Abstract (web summary)

High-order optical nonlinearities are a key tool in photonics and quantum optics, but their use is hindered by materials’ small intrinsic high-order susceptibility. Here, the authors show how to realize high-order nonlinear processes by combining intrinsic low-order ones in a microcavity.

## Introduction

Since the invention of lasers, a wide range of nonlinear optics processes have been experimentally observed in dielectric materials and have deepened our understanding of the physics of light-matter interactions^1–5^. Nonlinear optics effects not only provide a unique testbed for studying nonlinear physics with flexible parameters over many orders, but also allow various applications, including frequency conversion for optical detection and imaging^6^, comb laser-based precision spectroscopy^7^, material characterization and bio-chemical sensing ^8^. For emerging quantum information science, optical nonlinearity is the key resource for nontrivial tasks enabled by quantum mechanics, ranging from communication, sensing to computation. Coherent multi-wave mixing allows the generation of entangled photon pairs^9^ and also quantum frequency conversion^10^ to connect distinct quantum systems. In particular, high-order nonlinearities are desired for the generation of exotic many-photon entangled states or the realization of controllable photon-photon quantum gates^11,12^, which are crucial for an extensible quantum system. Nonetheless, most studies are limited to the low-order nonlinearity of dielectric materials, i.e., the *χ*^(2)^ and *χ*^(3)^ processes, because the nonlinear susceptibility decays exponentially with the order. To date, the fundamental physics of high-order optical nonlinearity and the associated applications are rarely investigated in experiments using solid-state materials^13,14^.

Recently, nonlinear photonic devices on integrated photonic chips^15–18^ have attracted tremendous research interest due to their advantages in compactness, stability, and low-power-consumption. Compared with conventional bulky nonlinear crystals and nonlinear fibers, integrated microcavity significantly enhances light-matter interaction due to the strongly confined mode volume *V*_m_ as well as the high-quality factor^19^. In a microcavity, the nonlinear coupling rate of an (*n* + 1)-wave mixing process scales as ${g}_{n}\propto {\chi }^{(n)}/{V}_{{{{{{{{\rm{m}}}}}}}}}^{\left(n-1\right)/2}$, with ${\chi }^{(n)} \sim {{{{{{{\mathcal{O}}}}}}}}\left(1{0}^{-10(n-1)}\,{{{{{{{{\rm{(V/m)}}}}}}}}}^{n-1}\right)$ being the nonlinear susceptibility^1^. Currently, nonlinear enhancement has reduced the optical parametric oscillation threshold dramatically to micro-Watts based on *χ*^(2)^ and *χ*^(3)^ processes^20,21^. However, the enhancement of *g*_*n*_ provided by the scaling factor ${({V}_{{{{{{{{\rm{m}}}}}}}}})}^{(n-1)/2}$ is usually on the order of 10^5(*n*−1)^ for widely studied integrated microcavities, which cannot compensate the 10 order decrease of high order *χ*^(*n*)^ against *n*. It is still challenging to directly realize the multi-wave mixing involving five or more photons based on the intrinsic nonlinear susceptibility of common materials, even in microcavities.

In this work, we demonstrate an approach to synthesize *χ*^(4)^ process by combining intrinsic low-order nonlinear processes in a microcavity. The synthetic *χ*^(4)^ nonlinearity is demonstrated by generating correlated photon pairs, which shows a rate over 500 times higher than that due to the intrinsic *χ*^(4)^ susceptibility of the material. Its coherent property is verified by measuring the two-photon quantum interference in the time domain, which manifests an entangled photon-pair source between the visible and telecom bands. Our synthetic five-wave mixing (5WM) approach only needs a single infrared (IR) pump laser, releasing the requirement of ultraviolet or specially designed laser wavelengths in low-order nonlinear processes^18,22^. The phase-matching condition is also released from three wavelength bands to two bands, reducing the difficulty in engineering photonic structures. In addition, since other high-order nonlinear processes can be constructed, the synthetic approaches thus show high flexibility in choosing the pump wavelengths releasing the difficulty of phase-matching. Our scheme points to a universal route to synthesize high-order nonlinear processes based on low-order nonlinear processes in a single microcavity and would stimulate future experimental investigations on even higher-order nonlinear processes and the preparation of multi-photon quantum states^23,24^.

## Results

### Synthetic five-wave mixing

**Fig. 1:**
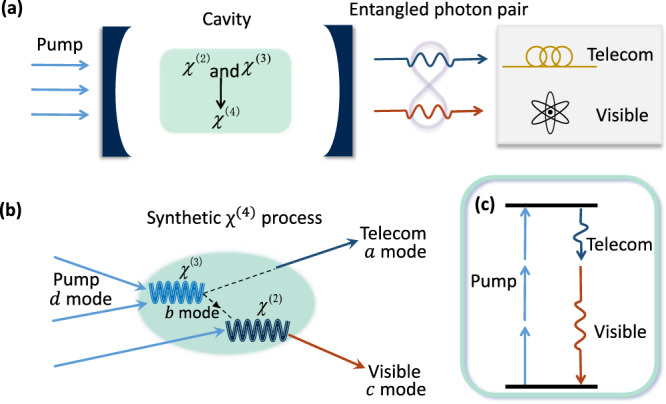
Schematic illustration of synthetic five-wave mixing. **a** Synthetic *χ*^(4)^ process constructed by cavity-enhanced *χ*^(2)^ and *χ*^(3)^ nonlinear processes for visible-telecom entangled photon-pair generation. **b**,**c** The scattering map and the equivalent energy level diagram of the synthetic *χ*^(4)^ process.

The principle of synthetic optical nonlinearity is illustrated in Fig. 1. In an optical cavity filled with non-centrosymmetric materials [Fig. 1(a)], the low-order nonlinear optical processes, i.e., three-wave mixing (3WM) and four-wave mixing (4WM) due to the material’s intrinsic *χ*^(2)^ and *χ*^(3)^ nonlinearities, respectively, could both be enhanced by the resonances. The two separate processes are independent of each other except sharing a common optical mode. For instance, as shown by the scattering map in Fig. 1(b), a photon generated by the 4WM could be a seed for the 3WM, and eventually an effective 5WM is synthesized by combining 4WM and 3WM. Denoting the shared mode for 4WM and 3WM as *b*, the four-photon and three-photon interactions could be described by the Hamiltonian as ${g}_{3}\left(ab{d}^{{{{\dagger}}} 2}+{a}^{{{{\dagger}}} }{b}^{{{{\dagger}}} }{d}^{2}\right)$ and ${g}_{2}\left({b}^{{{{\dagger}}} }{d}^{{{{\dagger}}} }c+bd{c}^{{{{\dagger}}} }\right)$, respectively, where *a*, *c*, *d* represent the other involved photonic modes, and *g*_2(3)_ represents the photon-photon interaction strength due to second (third)-order nonlinearity. When accessing the system only through modes *a*, *c* and *d*, and by treating the intermediate photon in *b* as virtual excitation, we could obtain the synthetic 5WM as

1 $$H={g}_{{{{{{{{\rm{4,eff}}}}}}}}}\left({d}^{{{{\dagger}}} 3}ac+{a}^{{{{\dagger}}} }{c}^{{{{\dagger}}} }{d}^{3}\right),$$

where *g*_4,eff_ = *g*_2_*g*_3_/Λ_*b*_ is the effective five-photon interaction strength and Λ_*b*_ is the equivalent detuning of the intermediate mode *b* [see Supplementary Note 3 for more details]. The synthetic 5WM can be used to construct the parametric interaction between telecom wavelength modes (*d* and *a*) and visible mode *c*: when driving *d*, a pair of photon could be generated in *a* and *c*, as depicted in Fig. 1(c). Compared with the material’s intrinsic *χ*^(4)^, the synthetic nonlinearity holds many advantages: First, the synthetic *g*_4,eff_ could be much higher than that from intrinsic *χ*^(4)^, thus enabling stronger multi-photon interactions. Second, elementary (low-order) process could be engineered individually and then combined for synthetic nonlinearity, thus the complicated dispersion engineering for high-order modes with poor modal overlaps is avoided for practical applications. It also provides an universal approach for constructing higher-order processes. For the example above, if we replace the *χ*^2^ process by another *χ*^3^ process ${g}_{3}^{\prime}(cb{e}^{{{{\dagger}}} 2}+{c}^{{{{\dagger}}} }{b}^{{{{\dagger}}} }{e}^{2})$ and keep *b* as the intermediate photon, six-wave mixing ${g}_{5,{{{{{{{\rm{eff}}}}}}}}}(c{d}^{2}{a}^{{{{\dagger}}} }{e}^{{{{\dagger}}} 2}+h.c.)$ could be realized (${g}_{5,{{{{{{{\rm{eff}}}}}}}}}\propto {g}_{3}{g}_{3}^{\prime}$) in a single device (see Supplementary Information), in contrast to the previous schemes by cascading multiple nonlinear optical components^25^.

### Experimental characterization

**Fig. 2:**
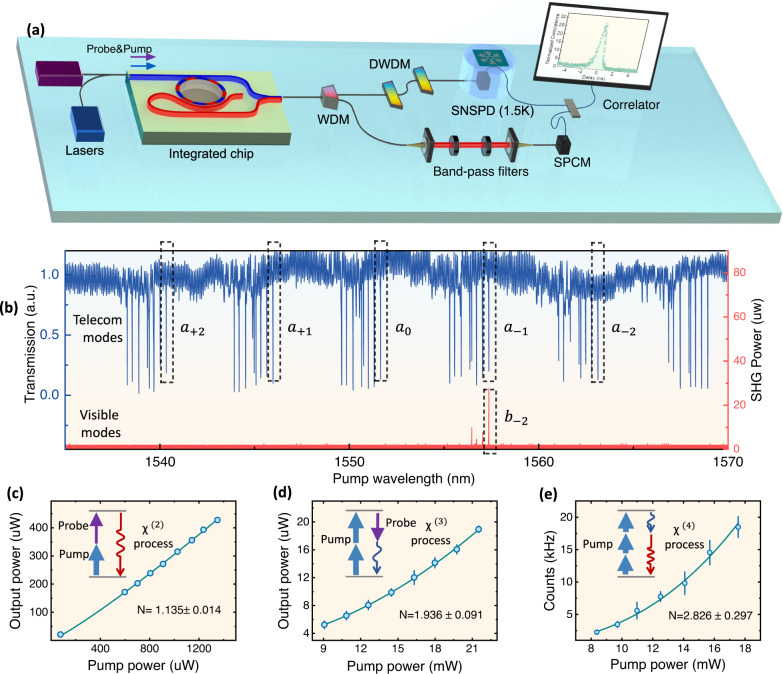
Characterization of the nonlinear process. **a** The experimental setup for 5WM. Telecom pump and probe lasers are coupled into the chip through a fiber lens and the output signals are collected by another fiber lens. The visible and telecom signal photons are separated by a wavelength division multiplexer (WDM), and then filtrated from background via a series of band-pass filters and dense wavelength division multiplexing (DWDM), respectively. The coincidence of generated photon pairs is characterized by single photon detectors (SPCM and SNSPD) and a correlator. **b** Transmission spectrum of telecom modes and the corresponding second-harmonic generation. The dips in dashed frames correspond to the modes characterized for synthetic five-wave mixing. The on-chip pump power is 1.8 mW. **c–e** The input-output relation of *χ*^(2)^, *χ*^(3)^ and *χ*^(4)^ processes. In **c** and **d**, we add a probe light at *a*_−2_ mode to construct stimulated *χ*^(2)^ and *χ*^(3)^ process and collect the output photon at *b*_−2_ and *a*_2_ separately. The on-chip probe power in **c** is 22 mW and in **d** is 1.1 mW. Error bars denote standard deviations.

The 5WM process is experimentally synthesized in a chip-integrated aluminum nitride microring, which provides excellent *χ*^(2)^ and *χ*^(3)^ properties and has been extensively studied in comb generation and high-efficient second-harmonic generation (SHG)^26–28^. Here, the effective *χ*^(4)^ is constructed between the fundamental TM_00_ modes at telecom wavelength (~1550 nm) and TM_20_ modes at visible wavelength (~775 nm). For the telecom modes only in a relatively narrow frequency range (~1550 ± 15 nm), the dispersion is negligible and all modes could participate in the 4WM efficiently. The microring was subsequently designed with an appropriate width to realize the phase-matching of 3WM between the TM_00_ telecom drive mode and TM_20_ visible mode^29^. Efficient sum-frequency generation (SFG) is realized by finely tuning the chip temperature near the SFG phase-matching point. As the experimental setup shows in Fig. 2(a), telecom drive lasers including pump and probe laser are injected into the device from one side of the chip through a fiber lens. The output signals are collected by another fiber lens on the other side of the chip, and are sequentially separated into different paths by a wavelength division multiplexer (WDM). After that, we use single photon counting modules (SPCM) to detect visible output signal, with the potential background noise filtered by a series of band-pass filters, while the telecom output signal transmitted through cascaded dense wavelength division multiplexings (DWDM) is detected by a superconducting nanowire single photon detector (SNSPD).

Figure 2 (b) shows the transmission spectrum of the telecom modes, where the modes belonging to the same mode family are marked by black frames. Along with the scanning of the laser, a strong peak in the visible mode is observed as the phase-matching condition between modes *a*_−1_ and *b*_−2_ is satisfied, which indicates the highly efficient SHG. Here, *a*_*i*_, *b*_*i*_ denote the bosonic operators of the optical modes, with the subscript $i\in {\mathbb{Z}}$ denoting the relative mode index of the modes. Due to the small dispersion of telecom modes *ω*_*a*,−2_ + *ω*_*a*,0_ ≈ 2*ω*_*a*,−1_, the SHG also implies an efficient non-degenerate 3WM process (i.e., SFG) ${H}_{{{{{{{{\rm{SFG}}}}}}}}}={g}_{2}({a}_{0}{a}_{-2}{b}_{-2}^{{{{\dagger}}} }+h.c.)$. Combining the SFG with the special 4WM process ${H}_{{{{{{{{\rm{4WM}}}}}}}}}={g}_{3}({a}_{0}{a}_{0}{a}_{+2}^{{{{\dagger}}} }{a}_{-2}^{{{{\dagger}}} }+h.c.)$, which shares the same telecom mode *a*_−2_, the desired 5WM between *a*_0_, *a*_+2_ and *b*_−2_ is constructed under fast dynamics of the intermediate mode *a*_−2_. This synthetic process consumes three pump photons (*a*_0_ mode) to produce a telecom-visible photon pair at *a*_+2_ and *b*_−2_, respectively.

Inside the AlN microcavity, all these nonlinear optical processes can be greatly enhanced due to the strong mode confinement and high quality factor. To ensure an efficient 5WM process, the elementary nonlinear processes are verified by pumping in the *a*_0_ and probing in the intermediate mode *a*_−2_, in which we add the probe light to stimulate the *χ*^(2)^ and *χ*^(3)^ processes and collect the output photons at *b*_−2_ and *a*_+2_ separately. According to *H*_3WM_ and *H*_4WM_, for a given probe power, the output powers of mode *b*_−2_ and *a*_+2_ scale linearly and quadratically with the pump power for 3WM and 4WM, respectively. Figure 2(c,d) show the relation between the output power from the signal mode and the pump power. By fitting the input-output relationship with ${P}_{{{{{{{{\rm{out}}}}}}}}}=A\times {P}_{{{{{{{{\rm{pump}}}}}}}}}^{N}$ with a fixed probe power, we get *N* = 1.135 ± 0.014 for mode *b*_−2_ and *N* = 1.936 ± 0.091 for mode *a*_+2_, which proves the efficient 3WM and 4WM associating with the intermediate mode. Then, by turning off the probe laser of the intermediate mode, the synthetic 5WM is tested using a coherent pump on the telecom mode *a*_0_, which produces an effective parametric Hamiltonian ${g}_{{{{{{{{\rm{eff}}}}}}}}}{n}_{a}^{3/2}({b}_{-2}{a}_{+2}+{b}_{-2}^{{{{\dagger}}} }{a}_{+2}^{{{{\dagger}}} })$ with an intracavity pump photon number *n*_*a*_. Such vacuum-induced photon-pair generation process results in a single-photon count rate of tens kilo-Herz from the visible mode *b*_−2_ by the SPCM. The power-dependent counts are fitted with *N* = 2.826 ± 0.30, agreeing well with the theoretical prediction of cubic power dependence ($\propto {n}_{a}^{3}$)and corresponding to the effective *g*_4_/2*π* ≈ 4.1 × 10^−5^ Hz.

### Visible-telecom entanglement

Although the cubic power dependence of the photon-pair rate demonstrates that five photons participate simultaneously in this 5WM process, it lacks direct evidence on the coherence of the synthetic nonlinear process, which is vital for potential quantum device applications in quantum information processing^30–32^, including the quantum frequency conversion and entangled photon sources. Therefore, the inherent coherent property of the synthetic *χ*^(4)^ process is further investigated by measuring the temporal correlation function and time-energy quantum entanglement between modes at the telecom band and the visible band, under the pump on mode *a*_0_ by a monochromatic laser.

**Fig. 3:**
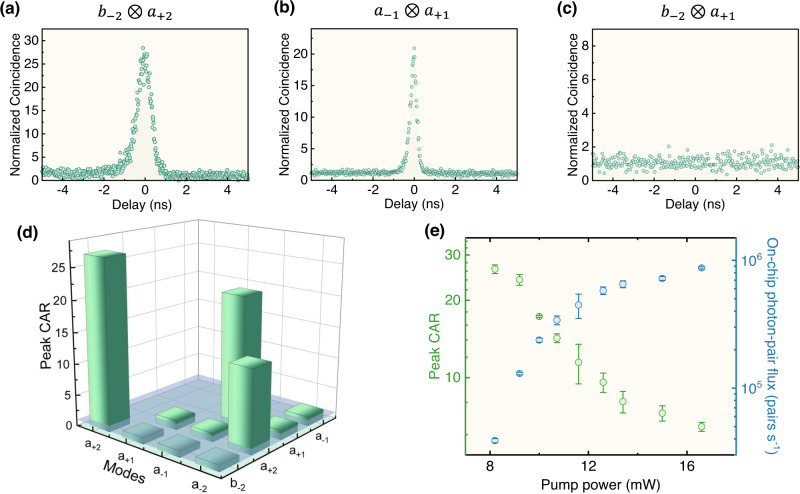
Photon-pair coincidence. **a–c** Normalized coincidence spectra for different mode pairs.The mean background levels (that is, accidental counting) are normalized to 1 and the normalized coincidence means the ratio of coincidence count to background. The on-chip pump power is 8.2 mW. **d** The peak coincidence to accidental rate (CAR) matrix of different mode pairs. The value of the blue reference flat denotes 1. **e** Pump power dependence of the peak CAR for visible--telecom photons (*b*_−2_⨂*a*_+2_) and on-chip photon-pair flux. Error bars denote standard deviations.

Figure 3 (a–c) show the normalized coincidence spectra for the visible-telecom correlation (*b*_−2_⨂*a*_+2_) and (*b*_−2_⨂*a*_+1_), as well as telecom-telecom correlation (*a*_−1_⨂*a*_+1_). It is found that only the 4WM and target 5WM produce the correlation, while the un-phase-matched interaction is excluded. Furthermore, the peak coincidence to accidental rates (CARs) between the involved modes are summarized in Fig. 3(d). The coincidence map shows that there are correlations between the *a*_+2_ mode and *a*_−2_ mode, *a*_−1_ mode and *a*_+1_ mode, *a*_+2_ mode and *b*_−2_ mode. The former two correlations correspond to the 4WM process and the third correlation correspond to the synthetic 5WM process. These results unambiguously exclude other potential multi-photon processes and noises. Note that for a Hermitian *χ*^(4)^-process, the photon generation in *a*_−2_ should be suppressed and thus the coincidence between *a*_−2_ and *a*_+2_ vanishes, which can be achieved in principle by independently engineering the resonance of mode *a*_−2_ far-off the phase-matching. The power dependence of the on-chip pair flux and peak CAR is shown in Fig. 3(e), with the on-chip photon-pair flux derived from the detected photon-pair flux by taking account of the losses of both signal and idler photons. As the pump power increases, the pair flux increases but the CAR value is limited by multi-pair generation in our process. While at the low pump region the decrease of pair flux is accompanied by the suppression of multi-pair events, so that CAR value is mainly limited by detector dark counts and imperfect filtering which is caused by the leakage of pump light at telecom band and pump SHG signal at visible band.

Furthermore, it can be inferred from Eq. (1) that a coherent quantum process enables the simultaneous generation of a pair of photons in target modes *b*_−2_ and *a*_+2_, instead of consequent realization of the 4WM and 3WM in a single microring. Comparing Fig. 1(a), (b), the temporal correlation function shows a similar symmetric profile, thus confirming that the synthetic nonlinearity resembles the intrinsic *χ*^(3)^ that generate photons simultaneously. Due to the higher dissipation rate of visible mode, the correlation function for *b*_−2_⨂*a*_+2_ shows a spread distribution of the generation time of the photon pairs, which can be characterized by coincidence measurements of different time offsets.

**Fig. 4:**
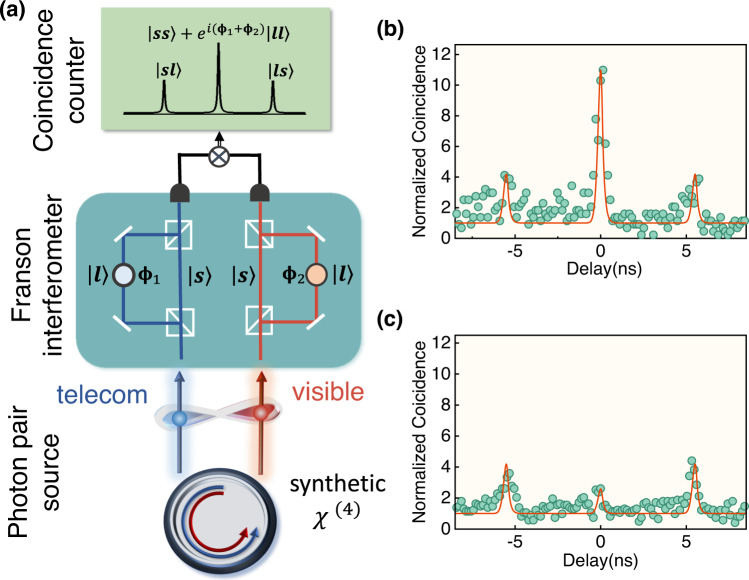
Visible-telecom entangled photon pair. **a** Characterization of the entanglement via Franson interferometer. There are two-unbalanced Mach--Zehnder interferometers (MZIs), for visible and telecom bands separately. The setup measures the interference between the short--short state ($\left|ss\right\rangle$) and long--long state ($\left|ll\right\rangle$), with the output coincidence depending on the phases of the MZIs (*ϕ*_1_ + *ϕ*_2_). **b**,**c** The interference fringes of the Franson interferometer, showing the constructive and destructive interference, with a peak CAR visibility of 72.7% ± 3.3%, corresponding *S* = 2.056 ± 0.093 for the Bell inequality test.

To verify the quantum coherence property of the synthetic *χ*^(4)^ and explore its potential applications, the time-energy entanglement between the emitted visible-telecom photon pairs is demonstrated. Through the two-unbalanced Mach-Zehnder interferometers (MZIs), the photon pairs are divided into four paths namely, short–short, long–long, short–long and long–short for visible–telecom channels, corresponding to twin-photon amplitude of different times. The time-energy entanglement is characterized via the Franson interferometer^33^. As shown at the top of Fig. 4(a), both the short-short and long-long twin-photon states contribute to the center peak of the coincidence spectrum, thus interference is expected for coherent parametric interaction. The equal time twin-photon quantum state can be expressed as: $\left|{{\Psi }}\right\rangle=\left|ss\right\rangle+{e}^{i({\phi }_{1}+{\phi }_{2})}\left|ll\right\rangle$, where $\left|ss\right\rangle$ and $\left|ll\right\rangle$ stand for twin photons from the short–short and long–long arms of the unbalanced MZI. The amplitude of the coincidence interference peak depends on the phases (*ϕ*_1_, *ϕ*_2_) of the two MZIs. In our experiment, we tune the phase *ϕ*_1_ + *ϕ*_2_ and track the coincidence spectrum. The remarkable change of the center peak in Fig. 4(b,c) demonstrates the existence of quantum interference. In particular, the visibility of the amplitude at the center peak achieves 72.7% ± 3.3%, corresponding *S* = 2.056 ± 0.093 for the Bell inequality test, which shows 72% confidence to violate the Bell’s inequality for a visible-telecom entangled photon-pair source.

## Discussion

Here, we have demonstrated synthetic 5WM in an integrated aluminum nitride microcavity. The coherent and quantum nature of the synthetic nonlinear process is validated by the quantum entanglement between visible and telecom photons, which are generated by a single telecom pump laser. It could be applied as quantum interfaces for future hybrid quantum network based on the Rubidium-atom quantum memories^34–36^. Comparing with the intrinsic *χ*^(4)^ nonlinearity of the material, our approach shows an enhancement of photon-pair generation rate by more than 500 times (see Supplementary Note 3 for derivation). In addition, coherent and fast tuning of the synthetic nonlinearity is enabled by controlling the intermediate mode, instead of the material property^37,38^. Remarkably, this synthetic 5WM also promises efficient on-chip three-photon sources by pumping at visible and telecom wavelengths, as a reversal of the process demonstrated here, and provides the Greenberger-Horne-Zeilinger resource states for photonic fusion-based quantum computing^39^.

It is anticipated that stronger *g*_4,eff_ and even higher-order synthetic nonlinearity (see Supplementary Note 3 for the general approach of synthetic nonlinearity) could be achieved in gallium arsenide, lithium niobate (LN) and indium gallium phosphide (InGaP) microcavities^40–43^. In particular, *g*_2_ approaching the 10 MHz-level, which is ~2 orders of magnitude higher than that of our current device, have been demonstrated with LN and InGaP and promise a significantly improved synthetic *χ*^(4)^. Moreover, our approach can be applied to all kinds of nonlinear platforms rather than limited in non-centrosymmetric materials with *χ*^(2)^ nonlinearity, and can be extended to hybrid systems, such as acoustic-optics involving photon-phonon interactions. Our demonstration opens the possibility of studying the fundamental physics in nonlinear multi-wave mixing and exploiting new functional quantum photonic devices.

## Methods

### Experimental device and setup

The aluminum nitride photonic chip used in our experiment is optimized for high-efficiency second-harmonic generation (SHG). The microring is designed with an appropriate width to realize phase-matching between the TM_00_ telecom drive mode and TM_20_ visible mode, and the exact frequency matching between visible and telecom modes is then realized by finely tuning the chip temperature. The radius of our device is ~30 μm with free spectrum range of ~700 GHz @1550 nm. Our device uses a straight bus waveguide to couple telecom light into the microrings and uses a specially-designed wrap-around waveguide for coupling visible light out.

In our experiment, the pump light is provided by amplifying the output of a telecom laser source (Agilent 8164A) with an erbium-doped optical fiber amplifier (EDFA, CONQUER, KG-EDFA-P). The amplified light is transmitted through a DWDM to filter the background noise caused by EDFA. The probe light is New Focus diode laser (TLB-6700) with a tunable laser controller. Our device is placed in an external heater (Covesion, PPLN Ovens-PV10) with a precise temperature controller (Covesion, OC2). For the filter system, we adopt multiple narrow band-pass filters (Semerock, LL01-780) for visible light, which can help us to filter the pump SHG signal and block background noise. In the telecom band, we use commercial 1550 nm band fiber DWDMs, with 100 GHz channel bandwidth.

### Single photon detection

Two kinds of single photon detectors were used to detect the generated visible and telecom single-photon-level signals separately. A superconducting nanowire single-photon detector (SNSPD, PHOTEC-1550) is used for telecom outputs with a high detection efficiency over 90%*@*1550 nm, a low dark count rate <100 Hz and a small jitter <20 ps in 1370–1680 nm band. The single photon counting modules (SPCM, Excelitas SPCM-NIR), with a photon detection efficiency of 70%*@*780 nm and a dark count rate of <200 Hz and is used for output in the visible band. For coincidence measurement between the modes, we use a high resolution time-to-digital converter (quTAG, standard 4 channels).The peak CAR values in Fig. 4 are calculated by CAR = (*C* − *A*)/*A*, where *C* and *A* are the overall and accidental coincidence counts obtained from the peak and background of the coincidence counting spectra. The one standard deviation uncertainty in Fig. 3(d) is given by multiple measurements.

### Franson interference

For the Franson interferometer, we use two-unbalanced MZIs for visible and telecom band signals, separately. The visible band unbalanced MZI is made up of spatial optical setup and uses a piezoelectric transducer to compensate for the phase drift due to the thermal effect and instability of the MZI via a proportion integration differentiation (PID) controller (SRS-SIM960). A fiber phase shifter and a PID controller are adopted to stabilize and adjust the phase at telecom band.

The interference visibility is calculated by the ratios extracted from the peak of coincidence spectra in Fig. 4(b,c) by ${{{{{{{\rm{Visibility}}}}}}}}=\frac{{{{{{{{{\rm{CAR}}}}}}}}}_{\max }-{{{{{{{{\rm{CAR}}}}}}}}}_{\min }}{{{{{{{{{\rm{CAR}}}}}}}}}_{\max }+{{{{{{{{\rm{CAR}}}}}}}}}_{\min }}$, where ${{{{{{{{\rm{CAR}}}}}}}}}_{\max }$ and ${{{{{{{{\rm{CAR}}}}}}}}}_{\min }$ are the center peak CAR values of the spectra. The one standard deviation uncertainty is given by $\frac{{\sigma }_{{{{{{{{\rm{CAR}}}}}}}}}}{{{{{{{{\rm{CAR}}}}}}}}}\approx \frac{1}{\sqrt{N}}$, *N* is the total coincidence counts at the peak, and the uncertainty of visibility is derived via the error propagation formula.

The interference visibility is limited by the signal-to-noise ratio of the single-photon outputs, which is mainly attributed to: (i) The low fiber-to-chip coupling efficiency for visible light, which limits the counts of the single photons at the visible wavelengths; (ii) The background noise due to the residue of the pump field and its second-harmonics. It can be improved with high-performance filters or by choosing the signal mode far from the pump in future experiments. (iii) Other parasitical nonlinear effects, such as Raman scattering.

## Supplementary information

Supplementary Information

## Data Availability

All data generated or analyzed during this study are available within the paper and its Supplementary Information. Further source data will be made available on request.

## Code availability

The code used to solve the equations presented in the Supplementary Information will be made available on request.
