# Supplementary material for: Synthetic five-wave mixing in an integrated microcavity for visible-telecom entanglement generation
Source: Nat Commun. 2022 Oct 20;13:6223. doi: 10.1038/s41467-022-33914-5 (PMC9585089; doi:10.1038/s41467-022-33914-5)
Supplement: Supplementary file 1 — Supplementary Information [file 41467_2022_33914_MOESM1_ESM.pdf]

Supplementary Information for "Synthetic five-wave mixing in an integrated microcavity for visible-telecom entanglement generation"

Jia-Qi Wang,[1,2,*] Yuan-Hao Yang,[1,2,*] Ming Li,[1,2,†] Haiqi Zhou,[1,2]
Xin-Biao Xu,[1,2] Ji-Zhe Zhang,[1,2] Chun-Hua Dong,[1,2] Guang-Can Guo,[1,2] C.-L. Zou,[1,2,†]
[1]CAS Key Laboratory of Quantum Information,
University of Science and Technology of China, Hefei 230026, China.
[2]CAS Center For Excellence in Quantum Information and Quantum Physics,
University of Science and Technology of China, Hefei 230026, China.
[*]These two authors contributed equally to this work.
[†]Corresponding author. E-mail: lmwin@ustc.edu.cn; clzou321@ustc.edu.cn;
(Dated: September 29, 2022)

SUPPLEMENTARY NOTE 1 - DEVICE PARAMETERS

The devices for this experiment are fully-etched AlN microstructures on silicon substrate, with a layer of silica in between. The geometry of waveguide is designed to use the intermodal dispersion to realize the phase-matching condition of the $\chi^{(2)}$ process between 1550 nm band and 775 nm band. More specifically, TM_{00} mode of IR band (1550 nm) and TM_{20} of visible band (775 nm) are selected for phase-matching. The radius of the microring is $30\,\mu m$, and the width of the bus waveguide is 570 nm. In order to achieve a large coupling rate between the mircoring and the bus waveguide for visible light, a thin waveguide wrapped around the ring is adapted to increase the effective coupling length. We select one of the device for investigating the synthetic five-wave mixing. The mode properties is characterized via the spectra for telecom input and second-harmonic generation, as shown in n Supplementary Figs. 1(c) and 1(d), which imply loaded quality factors of the device as 1.81×10^5 for the IR mode and 2.07×10^5 for the 775 nm (SHG) mode.

Considering the practical fabrication imperfections and variation of AlN film thickness, arrays of microring devices are fabricated to scan geometry parameters, thus the phase matching condition for the desired wavelengths is guaranteed for certain devices. As shown in n Supplementary Fig. 1(a), there are 16 microring devices in each array, with descending width of the mircoring waveguides from $1.15\,\mu$m to $1.25\,\mu$m. Combing fine temperature tuning, this method offers a high opportunity to select a microring satisfying the phase-matching condition of the $\chi^{(2)}$ process. Besides, the phase-matching condition of the $\chi^{(3)}$ process in 1560 nm band can be easily satisfied simultaneously due to a relatively small wavelength difference and small mode dispersion.

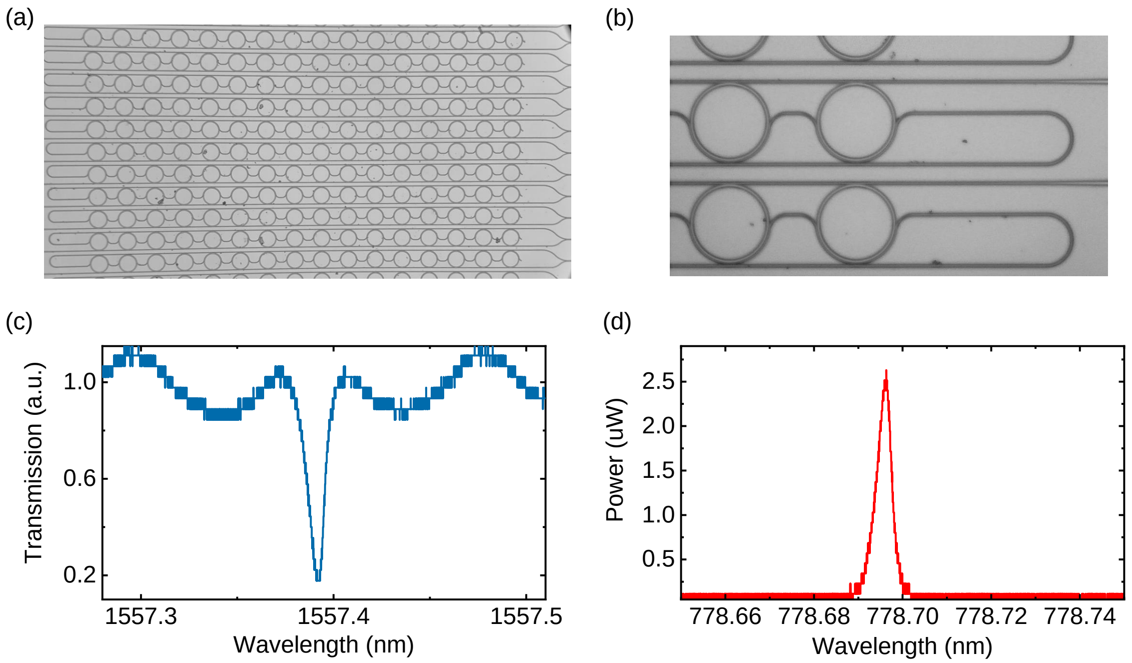

Supplementary Figure 1. **The devices. a**,**b**, The microring array structure and wrap-around design of the chip under the mircoscope. **c**,**d**, The transmission spectrum of the IR mode around 1557.40 nm, and the corresponding output signal in visible band via SHG process when the laser is scanning across the IR mode in (c).

SUPPLEMENTARY NOTE 2 - $\chi^{(2)}$ AND $\chi^{(3)}$ NONLINEARITY IN A MICRORING

A. Phase-matching condition

In order to allow for high efficient frequency conversion in nonlinear materials, the phase-matching condition must be fulfilled, which includes momentum conservation condition and energy conservation condition. For example, for a second harmonic process, the phase-matching condition implies that the wave-vectors of the pumping mode and the second harmonic (SH) mode at twice the frequency must be fulfilled. The condition is equivalent to the effective indices of the pump and the SH mode

must be equal. Because of the material dispersion, we cannot choose the fundamental mode in both pump mode and generated SH mode directly. So we need to engineer the geometry of the microring for modal dispersion to compensate for the material dispersion, which means that a higher-order mode in the SH wavelength needs to be used. In our system, we use TM_{00} mode of IR band and TM_{20} of visible band for phase-matching.

Therefore, for visible-IR photon-pair generation in conventional schemes based on spontaneous parametric down-conversion, it can be inferred that $\omega_(p,i,s)$ of the light locate at three distinct wavelength bands. we need to design a microring supports three band modes that satisfying the phase matching condition, and their frequency difference should be small when comparing with the optical mode linewidth. Since the refractive index of materials changes with the photon wavelength (dispersion), this requirement is very challenging for device design. In comparison, for our approach, the pump at 1550 nm is designed at the same band with the idler photon, thus the three-band dispersion engineering is released to two-band (780 nm and 1550 nm). Such an approach is much easier for experiments, as demonstrated in our work. On the other hand, based on the mechanism of synthetic nonlinearity, other high-order nonlinear process can be constructed so that a desired relationship between ω_p and $\omega_(s,i)$ can be built, releasing the difficulty on phase-matching and avoiding unusual laser frequency.

B. Measurement of $\chi^{(2)}$ and $\chi^{(3)}$ processes separately

In our experiment, the efficiency of individual $\chi^{(2)}$ and $\chi^{(3)}$ processes is characterized by tuning the phase-matching condition of the $\chi^{(2)}$ process. The phase-matching of degenerate four-wave mixing between a_0 and $a_{\pm 2}$ is very robust against temperature, since the frequency differences between these modes are relatively small (only approximately 20 nanometers), thus the phase-matching condition could be fulfilled as the FSR for these modes are almost identical and insensitive to the change of chip temperature. In contrast, the relative frequency difference for the $\chi^{(2)}$ process could effectively tuned by changing the chip temperature, since the mode frequencies are different by hundreds of nanometers and their mode families are also different.

Therefore, in our system, we measured the individual $\chi^{(3)}$ process by tuning the temperature of our chip to change the relative mode frequency detuning of the $\chi^{(2)}$ process, by which the $\chi^{(2)}$ process is effectively turned off. For the measurement of the $\chi^{(2)}$ process, the influence from the $\chi^{(3)}$ process cannot be eliminated due to the robust phase-matching of degenerate four-wave mixing between a_0 and a_{-2}. To avoid mutual influence, we design pump-probe experiments and work in the weak pump regime. With a pump on mode a_0, the probe signal on mode a_{-2} is translated to mode b_{-2}; Meanwhile it is also parametrically amplified through the $\chi^{(3)}$ interaction, which might cause an overestimation of the $\chi^{(2)}$ efficiency. According to our experimental results in Fig. 2(c), the efficiency of SFG depends linearly on the pump power, which agrees with the prediction for a pure $\chi^{(2)}$ process. The $\chi^{(3)}$-assisted parametric amplification and its output at 780 nm should quadratically depend on the pump power, and is negligible in this weak-pump measurement according to our previous work [1]. The quadratic relationship obviously deviates from our experimental observations of linear dependence. Therefore, for weak pump power, the influence of $\chi^{(3)}$ on the $\chi^{(2)}$ process can be safely neglected. From these measurements, the nonlinear coupling strengths $g_{2,3}$ can be quantified, and these coupling strengths are also insensitive to the chip temperature.

SUPPLEMENTARY NOTE 3 - THEORETICAL DERIVATIONS

C. General treatment

Without loss of generality, we consider two nonlinear processes that share a common mode. The total Hamiltonian of the system reads

$$H = \sum_j \omega_j O_j^\dagger O_j + g_1 \left(A^\dagger b + b^\dagger A\right) + g_2 \left(B^\dagger b + b^\dagger B\right), \tag{1}$$

where O_j is the photonic mode involved in the interaction, $g_{1,2}$ is the nonlinear coupling strength, A and B are tensor products of O_j or $O_j^\dagger$, depending on the form of nonlinear process. For example, A (B) can be O^2 for second-harmonic generation and $O_1 O_2$ for sum frequency generation. We can move to the rotating frame of the phase-matched interaction $A^\dagger B + AB^\dagger$ and obtain the interaction Hamiltonian

$$H_I = \left(g_1 A^\dagger + g_2 B^\dagger\right) b e^{-i\Delta t} + \left(g_1 A + g_2 B\right) b^\dagger e^{i\Delta t}, \tag{2}$$

where Δ is the detuning between operator A and B. Under large detuning Δ, rotating-wave approximation can be applied to get a time-independent interaction. To the second-order approximation, we can follow the approach in this book [2] and derive the

effective Hamiltonian as

$$H_{\mathrm{eff}} = \frac{1}{\Delta}\left[(g_1A+g_2B)\,b^\dagger,\left(g_1A^\dagger+g_2B^\dagger\right)b\right]$$
$$= -\frac{g_1^2}{\Delta}AA^\dagger - \frac{g_2^2}{\Delta}BB^\dagger - \frac{g_1g_2}{\Delta}AB^\dagger - \frac{g_1g_2}{\Delta}BA^\dagger$$
$$+\frac{g_1^2}{\Delta}\left[A,A^\dagger\right]b^\dagger b + \frac{g_2^2}{\Delta}\left[B,B^\dagger\right]b^\dagger b + \frac{g_1g_2}{\Delta}\left[A,B^\dagger\right]b^\dagger b + \frac{g_1g_2}{\Delta}\left[B,A^\dagger\right]b^\dagger b. \tag{3}$$

When the system is excited via the operators in A,B, the photon population in mode b is much smaller than these in other modes, thus the terms in the last line can be safely neglected. As the effects of the Hermitian terms $AA^\dagger$ and $BB^\dagger$ are to shift the mode resonance and $\frac{g_{1,2}^2}{\Delta}$ are usually much smaller than the mode dissipation, we arrive at the nontrivial synthetic nonlinearity $-\frac{g_1g_2}{\Delta}\left(AB^\dagger+BA^\dagger\right)$. The above analysis is general for any nonlinear processes. Based on the low-order nonlinearity, rich types of nonlinear processes can be synthesized.

D. 5WM

In our experiments, the microcavity posses both $\chi^{(2)}$ and $\chi^{(3)}$ nonlinearities. By finely tuning the temperature of the microring, the optimal phase-matching condition of the χ^2 process can be tuned for either second-harmonic generation (SHG) or sum-frequency generation (SFG) between different IR-visible mode pairs. To construct a five-wave mixing process, we employ both $\chi^{(2)}$ and $\chi^{(3)}$ processes in the microcavity, with the system total Hamiltonian reads

$$H_{\mathrm{tot}} = \omega_0a_0^\dagger a_0 + \omega_2a_2^\dagger a_2 + \omega_{-2}a_{-2}^\dagger a_{-2} + \Omega_{-2}b_{-2}^\dagger b_{-2}$$
$$+g_2(a_0a_{-2}b_{-2}^\dagger + a_0^\dagger a_{-2}^\dagger b_{-2}) + g_3(a_0a_0a_2^\dagger a_{-2}^\dagger + a_0^\dagger a_0^\dagger a_2a_{-2}) + \varepsilon_{\mathrm{p}}(a_0^\dagger e^{-i\omega_{\mathrm{p}}t} + a_0e^{i\omega_{\mathrm{p}}t}), \tag{4}$$

where $\varepsilon_{\mathrm{p}} = \sqrt{2\kappa_{a,1}P_{\mathrm{p}}/\hbar\omega_{\mathrm{p}}}$ denotes the pump field amplitude on mode a_0 and P_{p} is pump power. a_i and b_i are the annihilation operators of the i-th modes of 1560 nm band and 780 nm band, as shown in Fig. 1 in the main text. The mode frequencies are given by $\omega_i = \omega_0 + id_1 + \frac{i^2}{2}d_2$ and $\Omega_i = \Omega_0 + iD_1 + \frac{i^2}{2}D_2$ for a_i and b_i respectively, with d_N and D_N referring to their N-th order dispersion coefficients, respectively. g_2 and g_3 are the vacuum photon-photon nonlinear coupling strengths due to the $\chi^{(2)}$ and $\chi^{(3)}$ nonlinearities.

In the rotating frame of $\omega_{\mathrm{p}}a_0^\dagger a_0 + (\omega_0+2d_1-\delta_a)a_2^\dagger a_2 + (\omega_0-2d_1-\delta_a)a_{-2}^\dagger a_{-2} + (\Omega_0-2d_1-\delta_b)b_{-2}^\dagger b_{-2}$, the Hamiltonian reduces to

$$H = \Delta_{a0}a_0^\dagger a_0 + \Delta_{a2}a_2^\dagger a_2 + \Delta_{a-2}a_{-2}^\dagger a_{-2} + \Delta_{b-2}b_{-2}^\dagger b_{-2}$$
$$+g_2(a_0a_{-2}b_{-2}^\dagger + a_0^\dagger a_{-2}^\dagger b_{-2}) + g_3(a_0a_0a_2^\dagger a_{-2}^\dagger + a_0^\dagger a_0^\dagger a_2a_{-2}) + i\varepsilon_{\mathrm{p}}(a_0^\dagger + a_0), \tag{5}$$

where $\delta_a = \omega_0 - \omega_{\mathrm{p}}$, $\delta_b = \Omega_0 - 2\omega_{\mathrm{p}}$, $\Delta_{an} = \delta_a + \frac{n^2}{2}d_2$, and $\Delta_{bn} = \delta_b + \frac{n^2}{2}D_2 + n(D_1-d_1)$. For weak pump power, the system stays below the $\chi^{(3)}$-based parametric oscillation threshold, thus the backaction to mode a_0 from other modes are negligible. Then mode a_0 can be solved independently as a harmonic oscillator. Denoting the amplitude of mode a_0 as α, we have

$$\frac{d}{dt}\alpha = (-i\Delta_{a0} - \kappa_a)\,\alpha - i\varepsilon_{\mathrm{p}}. \tag{6}$$

At steady state, the solution is $\alpha = \frac{i\varepsilon_{\mathrm{p}}}{-i\Delta_{a0}-\kappa_a}$. Combining this result with the above Hamiltonian, we arrive at the reduced Hamiltonian for the system

$$H = \Delta_{a2}a_2^\dagger a_2 + \Delta_{a-2}a_{-2}^\dagger a_{-2} + \Delta_{b-2}b_{-2}^\dagger b_{-2}$$
$$+ g_2(\alpha a_{-2}b_{-2}^\dagger + \alpha^* a_{-2}^\dagger b_{-2}) + g_3(\alpha^2a_2^\dagger a_{-2}^\dagger + (\alpha^*)^2a_2a_{-2}). \tag{7}$$

Here the operators $b_{-2}, a_2^\dagger$ and a_{-2} correspond to operators A,B and b in the general analysis. By eliminating the intermediate excitation in a_{-2} under the large-detuning condition, we obtain the effective Hamiltonian

$$H_{\mathrm{eff}} = \Delta_{a2}a_2^\dagger a_2 + \Delta_{b-2}b_{-2}^\dagger b_{-2} + g_{4,\mathrm{eff}}\left(\alpha^3b_{-2}^\dagger a_2^\dagger + \alpha^{*,3}b_{-2}a_2\right). \tag{8}$$

The equation resembles the parametric generation of photon pairs in modes a_2 and b_{-2}, indicating an effective five-wave mixing that absorbing three pump photons and generating a photon pair or vice verca.

E. 6WM

Our approach to synthesize optical nonlinearities is universal and also is experimentally feasible by avoiding complicated dispersion engineering. The idea could be easily generalized to n-wave mixing process. For example, according to the general theory of nonlinearity synthesis, an effective 6-wave mixing (6WM) can be obtained by choosing two $\chi^{(3)}$ processes sharing one common mode. For example, assuming $A = ad^{\dagger 2}$ and $B = ce^{\dagger 2}$, we can get the interaction Hamiltonian of the 6WM process:

$$H_{\text{int}} = g_3(ad^{\dagger 2}b + a^\dagger d^2 b^\dagger) + g_3'(ce^{\dagger 2}b + c^\dagger e^2 b^\dagger)$$

and by treating the intermediate photon in b as intermediate excitation, we could obtain the effective Hamiltonian for synthetic six-wave mixing:

$$H_{\text{int}} = g_{5,\text{eff}}\left(cd^2 a^\dagger e^{\dagger 2} + h.c.\right)$$

Here, $g_{5,\text{eff}} \propto g_3 g_3'$ is the effective six-photon interaction strength.

F. Effective coupling strength $g_{4,\text{eff}}$ of the 5WM process

In presence of the cavity loss κ, the dynamics of the system state ρ follows the Lindblad master equation

$$\frac{d}{dt}\rho = -i[H,\rho] + \sum L_i[\rho], \tag{9}$$

where $L_i[\rho] = \kappa_i\left(2c_i\rho c_i^\dagger - c_i^\dagger c_i\rho - \rho c_i^\dagger c_i\right)$. The master equation is expanded as

$$\begin{aligned}
\frac{d}{dt}\rho =& -i\Delta_{a2}\left[a_2^\dagger a_2,\rho\right] - i\Delta_{a-2}\left[a_{-2}^\dagger a_{-2},\rho\right] - i\Delta_{b-2}\left[b_{-2}^\dagger b_{-2},\rho\right] - ig_2\left[\alpha a_{-2}b_{-2}^\dagger + \alpha a_{-2}^\dagger b_{-2},\rho\right] - ig_3[\alpha^2 a_2^\dagger a_{-2}^\dagger + (\alpha^*)^2 a_2 a_{-2},\rho] \\
&+\kappa_{a2}\left(2a_2\rho a_2^\dagger - a_2^\dagger a_2\rho - \rho a_2^\dagger a_2\right) + \kappa_{a-2}\left(2a_{-2}\rho a_{-2}^\dagger - a_{-2}^\dagger a_{-2}\rho - \rho a_{-2}^\dagger a_{-2}\right) + \kappa_{b-2}\left(2b_{-2}\rho b_{-2}^\dagger - b_{-2}^\dagger b_{-2}\rho - \rho b_{-2}^\dagger b_{-2}\right) \\
=& \Delta_{a2}' a_2^\dagger a_2\rho + \Delta_{a2}'^* \rho a_2^\dagger a_2 + 2\kappa_{a2}a_2\rho a_2^\dagger + \Delta_{a-2}' a_{-2}^\dagger a_{-2}\rho + \Delta_{a-2}'^* \rho a_{-2}^\dagger a_{-2} + 2\kappa_{a-2}a_{-2}\rho a_{-2}^\dagger \\
&+\Delta_{b-2}' b_{-2}^\dagger b_{-2}\rho + \Delta_{b-2}'^* \rho b_{-2}^\dagger b_{-2} + 2\kappa_{b-2}b_{-2}\rho b_{-2}^\dagger - ig_2\left[\alpha a_{-2}b_{-2}^\dagger + \alpha^* a_{-2}^\dagger b_{-2},\rho\right] - ig_3[\alpha^2 a_2^\dagger a_{-2}^\dagger + (\alpha^*)^2 a_2 a_{-2},\rho],
\end{aligned}$$

where $\Delta_j' = -i\Delta_j - \kappa_j$. In the Fock state basis, the density matrix element $\rho_{lmn,l'm'n'} =_{b_{-2},a_{-2},a_2} \langle n,m,l|\rho|l',m',n'\rangle_{a_2,a_{-2},b_{-2}}$. The master equation transform to

$$\begin{aligned}
\frac{d}{dt}\rho_{lmn,l'm'n'} =& -i(l-l')\Delta_{a_2}\rho_{lmn,l'm'n'} - (l+l')\kappa_{a_2}\rho_{lmn,l'm'n'} + 2\kappa_{a_2}\sqrt{(l+1)(l'+1)}\rho_{(l+1)mn,(l'+1)m'n'} \\
&-i(m-m')\Delta_{a_{-2}}\rho_{lmn,l'm'n'} - (m+m')\kappa_{a_{-2}}\rho_{lmn,lm'n'} + 2\kappa_{a_{-2}}\sqrt{(m+1)(m'+1)}\rho_{l(m+1)n,l'(m'+1)n'} \\
&-i(n-n')\Delta_{b_{-2}}\rho_{lmn,l'm'n'} - (n+n')\kappa_{b_{-2}}\rho_{lmn,lm'n'} + 2\kappa_{b_{-2}}\sqrt{(n+1)(n'+1)}\rho_{lm(n+1),l'm'(n'+1)} \\
&-ig_2(\alpha\sqrt{(m+1)n}\rho_{l(m+1)(n-1),l'm'n'} + \alpha^*\sqrt{m(n+1)}\rho_{l(m-1)(n+1),l'm'n'}) \\
&+ig_2(\alpha\sqrt{m'(n'+1)}\rho_{lmn,l'(m'-1)(n'+1)} + \alpha^*\sqrt{(m'+1)n'}\rho_{lmn,l'(m'+1)(n'-1)}) \\
&-ig_3(\alpha^2\sqrt{lm}\rho_{(l-1)(m-1)n,l'm'n'} + (\alpha^*)^2\sqrt{(l+1)(m+1)}\rho_{(l+1)(m+1)n,l'm'n'}) \\
&+ig_3(\alpha^2\sqrt{(l'+1)(m'+1)}\rho_{lmn,(l'+1)(m'+1)n'} + (\alpha^*)^2\sqrt{l'm'}\rho_{lmn,(l'-1)(m'-1)n}).
\end{aligned}$$

For weak pump, the quantum state of the system can be expanded by the truncated Fock-state space $\{|000\rangle,|100\rangle,|010\rangle,|001\rangle,|110\rangle,|101\rangle\}$. Submit the state basis into the dynamics of ρ, and assume $\kappa_{a_2} = \kappa_{a_{-2}} = \kappa_a$ in nondepletion approximation, we can obtain steady-state introcavity two photon population:

$$\rho_{101,101} = \frac{\alpha^6 g_3^2 g_2^2\left(\Delta_{a_{-2}}^2 + \Delta_{a_2}(\Delta_{a_{-2}} - \Delta_{b_{-2}}) - \Delta_{a_{-2}}\Delta_{b_{-2}} + (3\kappa_a+\kappa_b)^2\right)}{(\kappa_a+\kappa_b)^2\left((\Delta_{a_{-2}} - \Delta_{b_{-2}})^2 + (3\kappa_a+\kappa_b)^2\right)\left((\Delta_{a_{-2}} + \Delta_{a_2})^2 + 4\kappa_a^2\right)}. \tag{10}$$

which is proportion to $(g_2 g_3)^2$.

We note that the photon-pair population $\rho_{101,101}$ can be also generated by Hamiltonian of the intrinsic 5WM process:

$$H_{\text{int}} = \omega_0 a_0^\dagger a_0 + \omega_2 a_2^\dagger a_2 + \Omega_{-2} b_{-2}^\dagger b_{-2} + g_{4,\text{int}}(a_0^3 a_2^\dagger b_{-2}^\dagger + a_0^{\dagger 3} a_2 b_{-2}) + \varepsilon_{\text{p}}(a_0^\dagger e^{-i\omega_{\text{p}}t} + a_0 e^{i\omega_{\text{p}}t}), \tag{11}$$

which gives a two-photon population

$$\rho_{101,101,\text{int}} = \frac{\alpha^6 g_{4,\text{int}}^2 \kappa_{a_2}\kappa_{b_{-2}}}{\alpha^6 g_{4,\text{int}}^2 \left(\kappa_{a_2}+\kappa_{b_{-2}}\right)^2 + \kappa_{a_2}\kappa_{b_{-2}}\left(\left(\kappa_{a_2}+\kappa_{b_{-2}}\right)^2 + \left(\Delta_{a_2}+\Delta_{b_{-2}}\right)^2\right)}$$
$$\approx \frac{\alpha^6 g_{4,\text{int}}^2}{\left(\kappa_{a_2}+\kappa_{b_{-2}}\right)^2 + \left(\Delta_{a_2}+\Delta_{b_{-2}}\right)^2}. \tag{12}$$

Here, $g_{4,\text{int}}$ is the coupling strength induced by the intrinsic fourth-order nonlinear susceptibility. Comparing $\rho_{101,101,\text{int}}$ with the population in Eq. (10), and neglect the influence from detuning of a_2 and b_{-2} mode we can derive effective coupling strength $g_{4,\text{eff}}$ induced by synthetic nonlinearity as

$$g_{4,\text{eff}} = \sqrt{\frac{g_3^2 g_2^2\left(\Delta_{a_{-2}}^2 + \Delta_{a_2}(\Delta_{a_{-2}} - \Delta_{b_{-2}}) - \Delta_{a_{-2}}\Delta_{b_{-2}} + (3\kappa_a+\kappa_b)^2\right)\left(\left(\kappa_{a_2}+\kappa_{b_{-2}}\right)^2 + \left(\Delta_{a_2}+\Delta_{b_{-2}}\right)^2\right)}{(\kappa_a+\kappa_b)^2\left((\Delta_{a_{-2}}-\Delta_{b_{-2}})^2 + (3\kappa_a+\kappa_b)^2\right)\left((\Delta_{a_{-2}}+\Delta_{a_2})^2 + 4\kappa_a^2\right)}} \tag{13}$$

$$\approx \sqrt{\frac{g_3^2 g_2^2}{\left(\Delta_{a_{-2}}^2 + 4\kappa_a^2\right)}} \tag{14}$$

Based on the experimental results of Figs.2(c)-(d) in the main text, the fitted coupling strength of the $\chi^{(2)}$ and $\chi^{(3)}$ processes are $g_2/2\pi = 7.9\times 10^4\,\text{Hz}$ and $g_3/2\pi = 0.98\,\text{Hz}$, respectively. Submitting these values into Eq. (14), we get the value of $g_{4,\text{eff}}$ under small detuning as $g_{4,\text{eff}}/2\pi \approx 5.8\times 10^{-5}\,\text{Hz}$, which is close to the fitting result (Figures.2(e)) $g_{4,\text{fit}}/2\pi \approx 4.1\times 10^{-5}\,\text{Hz}$.

We note that the rotating-wave approximation on Eq.(2) is performed without considering the dissipation of the modes. However, for an open quantum system, there is also energy leakage through the intermediate mode. In this case, even though the Hamiltonian of the synthetic nonlinearity maintains the same form, the effective coupling coefficient $g_{4,\text{eff}}$ could be a complex number [3], and an additional nonlinear dissipation operator a_2 arises, which indicates a non-Hermitian 5WM process and more interesting physics on engineered nonlinear dissipation. On the one hand, the dissipation-modified coupling coefficient will decrease the photon-pair generation rate compared to the mode a_{-2} with high-Q. On the other hand, the incidental nonlinear dissipation can also be engineered to a target form by designing the synthetic process, which might be useful for stabilization or generation of photonic quantum states.

G. Estimation for $g_{4,\text{int}}$ induced by intrinsic nonlinear susceptibility

The intrinsic material $\chi^{(4)}$ would also lead to the same five wave fixing process, which is allowed in the same microring as we designed for synthetic $\chi^{(4)}$ based on $\chi^{(2)}$ and $\chi^{(3)}$ processes. The nonlinear coupling strength for the system inside the microring, which is denoted as $g_{4,\text{int}}$, can be derived as

$$\hbar g_{4,\text{int}} = \varepsilon_0 \iiint \mathrm{d}\theta r \mathrm{d}r \mathrm{d}z \frac{5\chi^{(4)}(r)}{2\sqrt{2}}\left[u_{a,0}^*(r)\right]^3 u_{a,2}(r) u_{b,-2}(r). \tag{15}$$

Here, $\chi^{(4)}$ is the intrinsic fourth-order susceptibility of the material and ε_0 is the vacuum permittivity. $u_{a(b),z}(r) = u_{a(b),i}(r,z)e^{im_{a(b),i}\theta}$ is the complex amplitude of the electrical field of optical mode $a(b)$, where $m_{a(b),i}$ is the angular momentum of mode $a(b)_i$. The normalization condition should be satisfied, which writes

$$\iiint \mathrm{d}\theta r \mathrm{d}r \mathrm{d}z \varepsilon_0 \varepsilon_r(\omega_{a(b),i}, r)|u_{a(b),i}(r)|^2 = \hbar\omega_{a(b),i}. \tag{16}$$

Here, ε_r is the relative dielectric permittivity. Introducing the effective mode-overlapping factor at the cross-section of the microring as

$$\zeta_4 = \frac{\iint \mathrm{d}r\mathrm{d}z\left[u_{a,0}^*(r)\right]^3 u_{a,2}(r) u_{b,-2}(r)}{\left[\iint \mathrm{d}r\mathrm{d}z|u_{a,0}(r)|^2\right]^{\frac{3}{2}}\sqrt{\iint \mathrm{d}r\mathrm{d}z|u_{a,2}(r)|^2 \iint \mathrm{d}r\mathrm{d}z|u_{b,-2}(r)|^2}}, \tag{17}$$

then we obtain the coupling strength

$$g_{4,\mathrm{int}} \approx \zeta_4 \left(\frac{\hbar}{\varepsilon_0 2\pi R}\right)^{\frac{3}{2}} \frac{\omega_a^2}{\varepsilon_a^2} \sqrt{\frac{\omega_b}{\varepsilon_b}} \frac{5\chi^{(4)}}{2\sqrt{2}} \times \delta[3m_a - (m_a+2) - (m_b-2)] \tag{18}$$

$$= \zeta_4 \left(\frac{\hbar}{\varepsilon_0 2\pi R}\right)^{\frac{3}{2}} \frac{\omega_a^2}{\varepsilon_a^2} \sqrt{\frac{\omega_b}{\varepsilon_b}} \frac{5\chi^{(4)}}{2\sqrt{2}} \times \delta(2m_a - m_b). \tag{19}$$

Note that several approximations are adapted here: (1) We assume that the radius of the microring is much larger than the width of the ring, and (2) $\chi^{(4)}$ and ε_r are nearly constant inside the cross-section of the microring. The integral over θ reduces to Kronecker delta function due to the symmetry, and only gives non-zero g_4 when the momentum conservation condition $2m_a - m_b = 0$ is satisfied.

Using the same method, we can derive the second- and third-order coupling strengths as

$$g_2 \approx \zeta_2 \left(\frac{\hbar}{\varepsilon_0 2\pi R}\right)^{\frac{1}{2}} \frac{\omega_a}{\varepsilon_a} \sqrt{\frac{\omega_b}{\varepsilon_b}} \frac{3\chi^{(2)}}{2\sqrt{2}} \times \delta(2m_a - m_b), \tag{20}$$

$$g_3 \approx \zeta_3 \frac{\hbar}{\varepsilon_0 2\pi R} \frac{\omega_a^2}{\varepsilon_a^2} \frac{3\chi^{(3)}}{4}, \tag{21}$$

where ζ_i denotes the mode-overlapping factor of the i-th order nonlinear process, with

$$\zeta_2 = \frac{\iint \mathrm{d}r\mathrm{d}z \left[u_{a,0}^*(r)\right]^2 u_{b,0}(r)}{\iint \mathrm{d}r\mathrm{d}z |u_{a,0}(r)|^2 \sqrt{\iint \mathrm{d}r\mathrm{d}z |u_{b,0}(r)|^2}}, \tag{22}$$

$$\zeta_3 = \frac{\iint \mathrm{d}r\mathrm{d}z \left[u_{a,0}^*(r)\right]^2 u_{a,2}(r) u_{a,-2}(r)}{\iint \mathrm{d}r\mathrm{d}z |u_{a,0}(r)|^2 \sqrt{\iint \mathrm{d}r\mathrm{d}z |u_{a,2}(r)|^2 \iint \mathrm{d}r\mathrm{d}z |u_{a,-2}(r)|^2}}. \tag{23}$$

Therefore, we could numerically calculate the second- and third-order susceptibility $\chi^{(2)}$ and $\chi^{(3)}$ according to the nonlinear coupling strength g_2 and g_3 that fitted from our experimental results. The result shows that $\chi^{(2)} = 3.04 \times 10^{-12}\,\mathrm{m/V}$, $\chi^{(3)} = 1.61 \times 10^{-21}\,\mathrm{m^2/V^2}$. According to the proportional relation between $\chi^{(2)}$, $\chi^{(3)}$ and $\chi^{(4)}$ [4, 5], we extrapolate the material's intrinsic fourth-order nonlinear susceptibility as

$$\chi^{(4)}_{\mathrm{extrapolate}} \approx 8.57 \times 10^{-31}\mathrm{m^3/V^3}. \tag{24}$$

Correspondingly, we expect an intrinsic fourth-order coupling strength $g_{4,\mathrm{int}}/2\pi = 2.40 \times 10^{-6}$Hz. With the same pump power, the enhancement factor for photon pair generation rate comparing the synthetic and intrinsic five wave mixing could be

$$\mathscr{E} = \left(\frac{g_{\mathrm{eff}}}{g_{\mathrm{int}}}\right)^2 = 584. \tag{25}$$

The enhancement factor for photon pair generation rate can be several orders of magnitude higher by improving the Q factor of the microcavity as well as selecting other materials with even higher $\chi^{(2)}$, i.e. thin film lithium niobate (TFLN). For potential applications of the synthetic nonlinearity in TFLN platform, we expect an $\mathscr{E} \approx 2 \times 10^7$ for $\chi^{(4)}$ with experimental parameters in Ref. [6].

SUPPLEMENTARY REFERENCES

[1] J.-Q. Wang, Y.-H. Yang, M. Li, X.-X. Hu, J. B. Surya, X.-B. Xu, C.-H. Dong, G.-C. Guo, H. X. Tang, and C.-L. Zou, "Efficient Frequency Conversion in a Degenerate χ^2 Microresonator," Phys. Rev. Lett. **126**, 133601 (2021).

[2] C. Gerry and P. Knight, *Introductory Quantum Optics* (Cambridge University Press, 2004).

[3] C.-W. Lee, C. Noh, and J. Kim, “Effective formalism for open-quantum-system dynamics: Time-coarse-graining approach,” Phys. Rev. A **97**, 012102 (2018).
[4] W. Ettoumi, Y. Petit, J. Kasparian, and J.-P. Wolf, “Generalized Miller Formulæ,” Opt. Express **18**, 6613 (2010).
[5] F. Bassani and V. Lucarini, “General properties of optical harmonic generation from a simple oscillator model,” Il Nuovo Cimento D **20**, 1117 (1998).
[6] J. Lu, M. Li, C.-L. Zou, A. Al Sayem, and H. X. Tang, “Toward 1% single-photon anharmonicity with periodically poled lithium niobate microring resonators,” Optica **7**, 1654 (2020).
